# *Poria cocos*-Derived Exosome-like Nanovesicles Alleviate Metabolic Dysfunction-Associated Fatty Liver Disease by Promoting Mitophagy and Inhibiting NLRP3 Inflammasome Activation

**DOI:** 10.3390/ijms26052253

**Published:** 2025-03-03

**Authors:** Tao Wang, Jun Zhao, Qiu-Yi Li, Hui-Qiong Yang, Min Li, Rong Duan, Mei Zhang, Yan Qi, Jie Yu, Xing-Xin Yang

**Affiliations:** 1 College of Pharmaceutical Science, Yunnan University of Chinese Medicine, 1076 Yuhua Road, Kunming 650500, China; 13330581606@163.com (T.W.); 18287498463@163.com (J.Z.); l384227168@163.com (Q.-Y.L.); 15752819630@163.com (H.-Q.Y.); lm08212000@163.com (M.L.); DuanRLucky@163.com (R.D.); meizhang213@163.com (M.Z.); qiyankm@163.com (Y.Q.); 2 Yunnan Key Laboratory of Southern Medicine Utilization, 1076 Yuhua Road, Kunming 650500, China

**Keywords:** *Poria cocos*-derived exosome-like nanovesicles, fatty liver, high-fat diet, inflammasome, mitophagy

## Abstract

Metabolic dysfunction-associated fatty liver disease (MAFLD) affects approximately one-quarter of the world’s adult population, and no effective therapeutic drugs are available. *Poria cocos* is a fungus used as a herb and food nutrient for centuries as well as for MAFLD treatment. Exosome-like nanovesicles have many pharmacological activities; however, studies on the effects of *Poria cocos*-derived exosome-like nanovesicles (PCELNs) on MAFLD are lacking. Therefore, our study aimed at identifying the effects and mechanism of action of PCELNs on MAFLD. PCELNs were isolated by ultracentrifugation and their morphology was characterized, such as particle size, zeta potential, protein distributions, as well as lipid and miRNA compositions. Then, the absorption and distribution of PCELNs were observed in vivo and in vitro. Finally, L02 cell steatosis model induced by fat emulsion and MAFLD mouse model induced by high-fat diet (HFD) were used to evaluate the effect and mechanism of PCELNs on MAFLD. PCELNs were membrane structured vesicles, with a particle size of 161.4 ± 1.7 nm, a zeta potential of −3.20 ± 0.37 mV, and contained a range of proteins, lipids, and miRNAs. PCELNs were absorbed by L02 cells and targeted the liver and spleen after intraperitoneal injection. PCELNs inhibited body weight gain and improved the index of heart, liver, spleen, and various fats, as well as decreased lipid accumulation and lipid level. They also protected mitochondrial ultrastructure and regulated oxidative stress and energy metabolism disorder. Furthermore, PCELNs increased PTEN induced kinase 1 (PINK1), E3 ubiquitin ligase (Parkin) and microtubule associated protein light chain-3 (LC3) protein expression in the liver, reduced oxidized mitochondrial DNA (Ox-mtDNA) content in mitochondria and cytoplasm of the liver, reduced nucleotide binding oligomerization domain-like receptor protein 3 (NLRP3), pro-cysteinyl aspartate specific proteinase-1 (caspase-1), cleared-caspase-1, and mature-interleukin-1β (IL-1β) protein expression in the liver, and reduced the levels of tumor necrosis factor-α (TNF-α), interleukin-6 (IL-6), IL-1β, and interleukin-18 (IL-18) in serum and liver. In conclusion, we demonstrated that PCELNs may alleviate HFD-induced MAFLD by promoting mitochondrial autophagy and inhibiting NLRP3 inflammasome activation.

## 1. Introduction

Metabolic dysfunction-associated fatty liver disease (MAFLD), formerly known as nonalcoholic fatty liver disease, is the most common chronic liver disease worldwide, with an estimated global prevalence of 25–30% [1,2]. MAFLD is closely related to metabolic disorders, such as obesity, hypertension, dyslipidemia, and type 2 diabetes [3]. Hepatic steatosis is a pathological hallmark of MAFLD, which can evolve into nonalcoholic steatohepatitis, fibrosis, cirrhosis, and hepatocellular carcinoma [4]. However, no currently approved drugs are available for the treatment of MAFLD. Therefore, it is of the utmost importance to develop safe and effective drugs to prevent and treat MAFLD.

*Poria cocos* (Schw.) Wolf (PC) is a fungus widely used as medicine and food nutrients in many countries, including China, South Korea, and Japan, as it has a cosmopolitan distribution [5]. PC has a long history as a traditional Chinese medicine, used for diuresis, dehumidification, invigorating the spleen, and tranquilizing the mind [6]. Modern pharmacological studies showed the presence of many constituents in PC, such as sterols, diterpenes, triterpenes, and polysaccharides, which protect the liver and have several beneficial effects, including anti-inflammatory, anti-tumor, antioxidant, and anti-hypoglycemic [5,6]. Extracts of PC regulate lipid metabolism and bile acid metabolism to alleviate MAFLD [7,8]. It also protects gut vascular barrier, thereby alleviating nonalcoholic steatohepatitis [9,10]. PC restores mitochondrial structure and function, alleviating MAFLD, which may be related to the 15 triterpenoids in PC [11]. Furthermore, PC polysaccharide reduces lipid levels in the serum and liver in patients with MAFLD, increases lipid use, and reduces lipid synthesis and absorption [12]. PC triterpenoids, such as poricoic acid B and polyporenic acid C, also have therapeutic effects on MAFLD [8].

Exosome-like nanovesicles (ELNs) are membrane-containing vesicles released in an evolutionarily conservative way from organisms including prokaryotes, higher eukaryotes, and plants [13]. Herb medicine-derived exosome-like nanovesicles (HELNs) allow cross-species communication [14]. They also show inherent biocompatibility, high modification flexibility, low immunogenicity, and intrinsic targeting ability. These characteristics give HELNs far-reaching application prospects in the treatment of diseases [15]. HELNs are a type of natural active substances containing proteins, lipids, nucleic acids, and metabolites [16], thus having a high pharmaceutical value, with anti-inflammatory, anti-tumor, and antibacterial activities, particularly in the treatment of cancer, gastrointestinal diseases, and liver diseases [14,15,16,17,18]. For example, *Panax ginseng*-derived nanoparticles inhibit melanoma growth by inducing macrophage polarization from M2 to M1 [19]. *Asparagus cochinchinensis*-derived ELNs enter hepatocellular carcinoma cells mainly through internalization and inhibit the proliferation of liver cancer cells [20]. *Catharanthus roseus*-derived ELNs target immune organs and alleviate cyclophosphamide-induced immunosuppression through the TNF-α/NF-κB/PU.1 axis [21]. *Portulaca oleracea*-derived ELNs target the inflammatory sites in mice with colitis, inhibit the expression of pro-inflammatory cytokines, and increase the level of the anti-inflammatory cytokine IL-10, thereby alleviating colitis [22].

This study described the first isolation of *Poria cocos*-derived exosome-like nanovesicles (PCELNs) by ultracentrifugation and performed a series of characterizations. PCELNs were taken up by L02 cells in vitro and selectively targeted the liver and spleen of mice in vivo. Furthermore, PCELNs effectively alleviated high-fat diet (HFD)-induced MAFLD by regulating lipid metabolism, oxidative stress, inflammatory response, and the function and quality of mitochondria by promoting PTEN induced kinase 1 (PINK1)/E3 ubiquitin ligase (Parkin)-mediated mitophagy and inhibiting the production and efflux of oxidized mitochondrial DNA (Ox-mtDNA), thereby inhibiting nucleotide binding oligomerization domain-like receptor protein 3 (NLRP3) inflammasome activation.

## 2. Results

### 2.1. Characteristics of PCELNs

**Figure 1:**
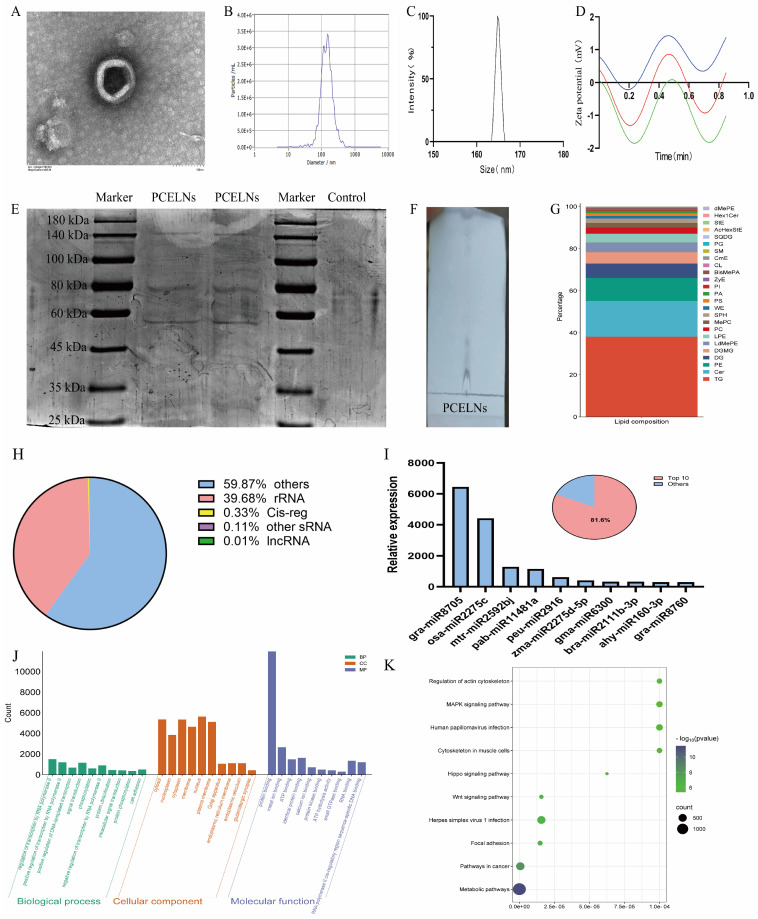
Physicochemical characterization and composition of PCELNs. (**A**) TEM image of PCELNs (scale bar = 100 nm). (**B**,**C**) Size distribution of PCELNs. (**D**) Zeta potential of PCELNs (Repeat the test three times). (**E**) Protein gel electrophoresis of PCELNs. (**F**) TLC image and (**G**) lipidomic analysis of PCELNs. (**H**) Classification of small nucleic acids contained in PCELNs. (**I**) Composition and proportion of top 10 miRNAs in PCELNs. (**J**) GO and (**K**) KEGG enrichment analysis of top 10 miRNAs in PCELNs. PCELNs, *Poria cocos*-derived exosome-like nanovesicles; TEM, transmission electron microscope; TLC, thin-layer chromatography; GO, Gene Ontology; KEGG, Kyoto encyclopedia of genes and genomes.

The size distribution and morphology of PCELNs were similar to those of ELNs as revealed by transmission electron microscope (TEM) (Figure 1A). Furthermore, the particle size of PCELNs was distributed in the 100 to 200 nm range, with an average particle size of 161.4 ± 1.7 nm, as revealed by dynamic light scattering (DLS) and nanoparticle tracking analyzer (NTA) analysis (Figure 1B,C). PCELNs showed a negative zeta potential of −3.20 ± 0.37 mV (Figure 1D), which contained a series of proteins that ranged widely in molecular size, from 25 kDa to 180 kDa (Figure 1E), and a range of lipids (Figure 1F). The lipidomic analysis indicated that PCELNs were mainly composed of triglyceride (TG, 38.13%), ceramide (Cer, 16.99%), phosphatidyl ethanolamine (PE, 10.87%), diacylglycerol (DG, 6.87%), and digalactosyl monoacylglycerol (DGMG, 5.39%) (Figure 1G). Subsequently, the types of small nucleic acids in PCELNs were assessed (Figure 1H) and miRNA sequencing technology was used to detect the miRNA spectra in PCELNs, revealing that the top 10 miRNAs accounted for 81.6% of the total miRNAs (Figure 1I). The two miRNAs, gra-miR-8705 and osa-miR-2275c, showed a high relative abundance, indicating their specific enrichment in PCELNs (Figure 1I). Furthermore, the targets of the top 10 miRNAs were divined by the algorithms of the public databases TargetScan and miRanda, which mainly involved the cellular component and the molecular function, as well as the regulation of the pathways in cancer and metabolic pathways (Figure 1J,K). However, the major active small molecules (sterols, diterpenes, and triterpenes) contained in PC were not found in PCELNs.

### 2.2. In Vitro Biocompatibility and Cytotoxicity of PCELNs

**Figure 2:**
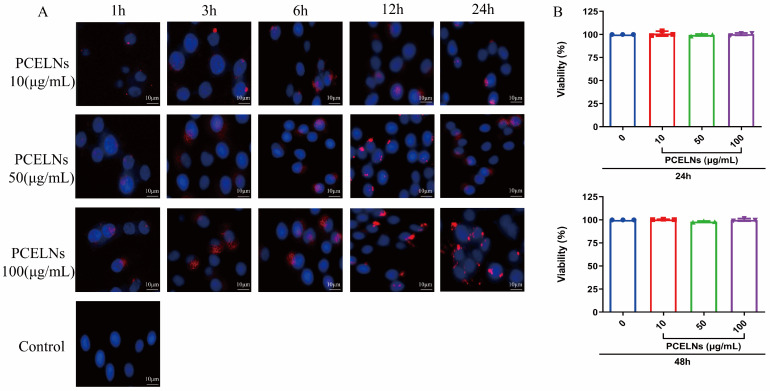
Biocompatibility and cytotoxicity of PCELNs. (**A**) Uptake of PCELNs by L02 cells at different times and concentrations (scale bar = 10 µm). (**B**) Cytotoxicity of PCELNs on L02 cells for 24 h and 48 h. *n* = 3. Results were expressed as mean ± S.D.

PCELNs were labeled with the red fluorescent dye PKH26 and incubated with L02 cells. The results showed that PCELNs were absorbed by L02 cells and were mainly located in the cytoplasm. The red fluorescence signal in L02 cells gradually enhanced with the increase in the incubation time and PCELN concentrations (Figure 2A). The assessment of the cytotoxicity of PCELNs on L02 cells revealed no significant change in the cell survival rate after incubation for 24 and 48 h with different concentrations of PCELNs (Figure 2B). Therefore, PCELNs were relatively safe nanovesicles.

### 2.3. In Vitro Anti-Steatotic Effects of PCELNs

**Figure 3:**
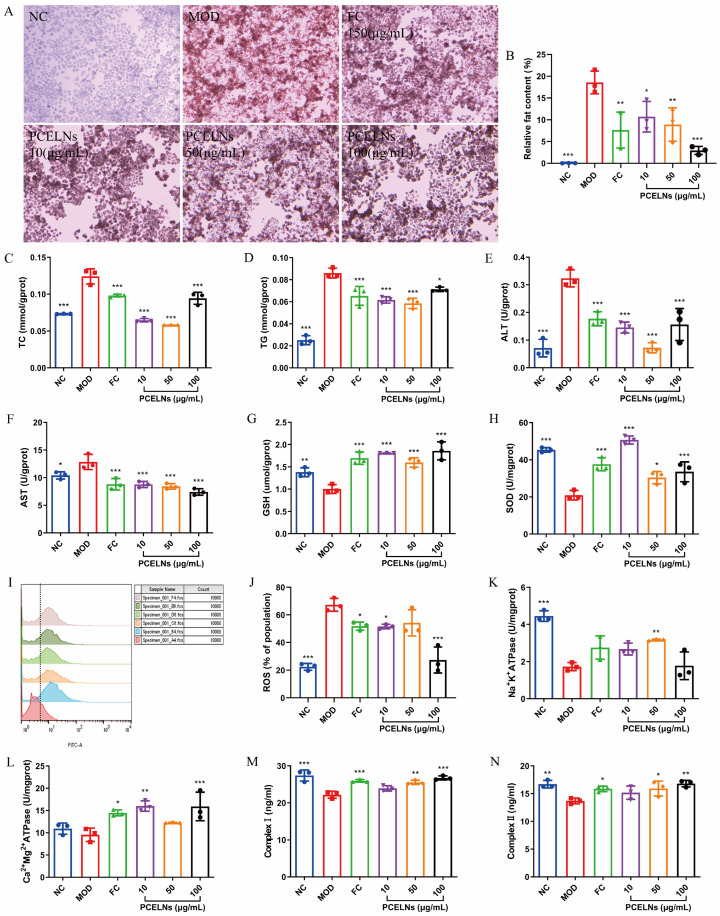
Effect of PCELNs on L02 cells. (**A**) Images of oil red O staining (scale bar = 100 µm). (**B**) Analysis of relative fat content. Cell levels of (**C**) TC, (**D**) TG, (**E**) ALT, (**F**) AST, (**G**) GSH, (**H**) SOD, (**I**,**J**) ROS, (**K**) Na^+^-K^+^-ATPase, (**L**) Ca^2+^-Mg^2+^-ATPase, (**M**) complex I, and (**N**) complex II. *n* = 3. Results were expressed as mean ± S.D. * *p* < 0.05, ** *p* < 0.01, *** *p* < 0.001 vs. MOD group. NC, normal control; MOD, high-fat diet; FC, fenofibrate capsules; TG, triglyceride; TC, total cholesterol; AST, aspartate transaminase; ALT, alanine transaminase; SOD, superoxide dismutase; GSH, glutathione; ROS, reactive oxygen species; ATPase, ATP synthase; complex I, respiratory chain complex I; complex II, respiratory chain complex II.

Oil red O staining was used to evaluate the degree of lipid accumulation. Moreover, 5% Fat emulsion induced lipid accumulation in L02 cells, which was significantly reversed after PCELN treatment (Figure 3A,B). Figure 3C–N shows that the levels of total cholesterol (TC), TG, alanine transaminase (ALT), aspartate transaminase (AST), and reactive oxygen species (ROS) were significantly increased (*p* < 0.05, *p* < 0.001), while the levels of glutathione (GSH), superoxide dismutase (SOD), Na^+^-K^+^-ATP synthase (ATPase), respiratory chain complex I (complex I), and respiratory chain complex II (complex II) were significantly decreased after 24 h incubation with 5% fat emulsion (*p* < 0.01, *p* < 0.001). The levels of Ca^2+^-Mg^2+^-ATPase showed a reduced trend. PCELN treatment for 24 h significantly decreased the levels of TC, TG, ALT, AST, and ROS (*p* < 0.05, *p* < 0.001), while the levels of GSH, SOD, Na^+^-K^+^-ATPase, Ca^2+^-Mg^2+^-ATPase, and complex I and II were significantly increased (*p* < 0.05, *p* < 0.01, *p* < 0.001). The above results indicated that PCELNs inhibited the steatosis of L02 cells induced by fat emulsion.

### 2.4. Effect of PCELNs on PINK1/Parkin-Mediated Mitophagy in L02 Cells

**Figure 4:**
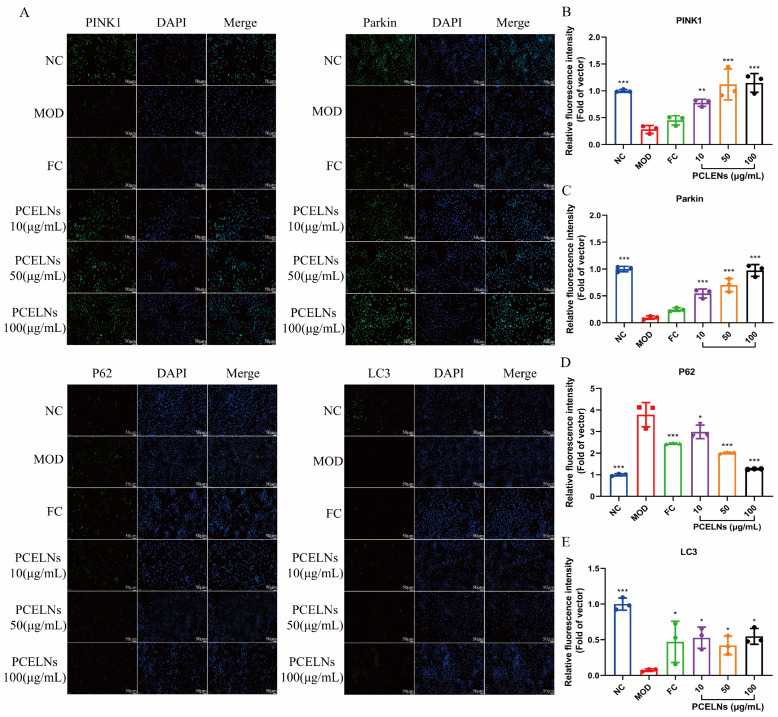
Effect of PCELNs on PINK1/Parkin-mediated mitophagy in L02 cells. (**A**) immunofluorescence images of PINK1, Parkin, LC3, and p62 (scale bar = 50 µm). Analysis of relative fluorescence intensity of (**B**) PINK1, (**C**) Parkin, (**D**) p62, and (**E**) LC3. *n* = 3. Results were expressed as mean ± S.D. * *p* < 0.05, ** *p* < 0.01, *** *p* < 0.001 vs. MOD group. PINK1, PTEN induced kinase 1; Parkin, E3 ubiquitin ligase; LC3, microtubule associated protein light chain-3; p62, sequestosome 1.

Related studies found that PINK1/Parkin-mediated mitophagy is inhibited in steatotic hepatocytes [23]. Therefore, the effect of PCELNs on the PINK1/Parkin signaling pathway was assessed by immunofluorescence. The results showed that sequestosome 1 (p62) protein expression increased, while PINK1, Parkin, and microtubule-associated protein light chain-3 (LC3) protein expression decreased after 24 h of induction with 5% fat emulsion. However, PINK1, Parkin, and LC3 protein expression increased, while p62 protein expression decreased after treatment for 24 h with PCELNs (Figure 4). This indicated that PCELNs mitigated steatosis in hepatocytes by promoting PINK1/Parkin-mediated mitophagy.

### 2.5. In Vivo Biodistribution of PCELNs

**Figure 5:**
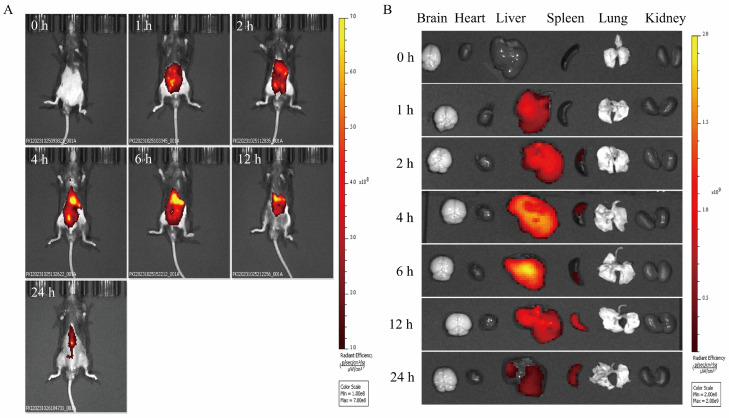
Biodistribution of PCELNs. (**A**) Biodistribution of PCELNs in mice after intraperitoneal injection. (**B**) Biodistribution of PCELNs in organs after intraperitoneal injection.

The distribution of PCELNs in mice was observed by the intraperitoneal injection of DIR-PCELNs. Different degrees of fluorescence signals were detected in mice at different time periods, and the fluorescence intensity at 4 and 6 h was the strongest, and then it decreased over time (Figure 5A). Mice tissues were imaged, showing fluorescent signals in the liver and spleen (Figure 5B). These results indicated that PCELNs were absorbed and used by mice and were mainly distributed in the liver and spleen.

### 2.6. In Vivo Anti-MAFLD Effects of PCELNs

#### 2.6.1. Effects of PCELNs on Body Weight, Food Intake, Organs, and Fat Index

**Figure 6:**
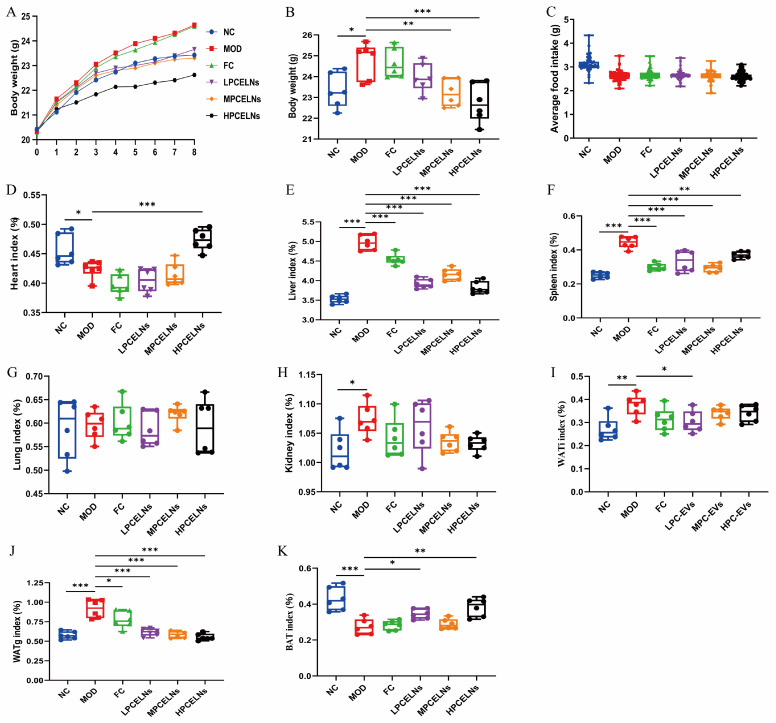
Effect of PCELNs on body weight, food intake, organs, and fat index. (**A**) Body weight. (**B**) Body weight in last week. (**C**) Average daily food intake. (**D**) Heart, (**E**) liver, (**F**) spleen, (**G**) lung, (**H**) kidney, (**I**) WATi, (**J**) WATg, and (**K**) BAT index. *n* = 6. Results were expressed as mean ± S.D. * *p* < 0.05, ** *p* < 0.01, *** *p* < 0.001 vs. MOD group. WATg, gonad white adipose tissue; WATi, inguinal white adipose tissue; BAT, brown adipose tissue; LPCELNs, low PCELN-treated group; MPCELNs, middle PCELN-treated group; HPCELNs, high PCELN-treated group.

A mouse model of MAFLD with HFD for 8 weeks and simultaneous administration of PCELNs at different doses was used to investigate the effect of PCELNs on body weight, organs, and fat of MAFLD. The body weight and the daily food intake of mice in each group recorded weekly (Figure 6A,C) revealed no significant difference in food intake among all groups. The body weight of mice in the last week revealed that HFD feeding caused a significant increase (*p* < 0.05) (Figure 6B). However, the body weight of mice was significantly decreased after treatment with PCELNs (*p* < 0.01, *p* < 0.001). The liver index, spleen index, kidney index, gonad white adipose tissue (WATg) index, and inguinal white adipose tissue (WATi) index of HFD-fed mice were significantly increased (*p* < 0.05, *p* < 0.01, *p* < 0.001), while the heart index and brown adipose tissue (BAT) index were significantly decreased (*p* < 0.05, *p* < 0.001) (Figure 6D–K). However, the heart index and BAT index of mice were significantly increased (*p* < 0.05, *p* < 0.01, *p* < 0.001), and the liver index, spleen index, WATg index, and WATi index of mice were significantly decreased after the treatment with PCELNs (*p* < 0.05, *p* < 0.01, *p* < 0.001). The above results indicated that PCELNs improved HFD-induced weight gain and changed some organs and fat indexes.

#### 2.6.2. Effects of PCELNs on Serum Lipid Parameters

**Figure 7:**
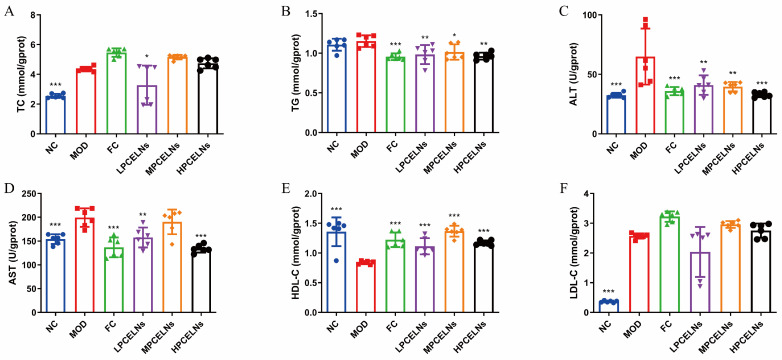
Effect of PCELNs on serum lipid-related parameters. Serum levels of (**A**) TC, (**B**) TG, (**C**) ALT, (**D**) AST, (**E**) HDL-C, (**F**) LDL-C. *n* = 6. Results were expressed as mean ± S.D. * *p* < 0.05, ** *p* < 0.01, *** *p* < 0.001 vs. MOD group. HDL-C, high-density lipoprotein cholesterol; LDL-C, low-density lipoprotein cholesterol.

MAFLD is usually accompanied by dyslipidemia. The levels of TC, ALT, AST, and low-density lipoprotein cholesterol (LDL-C) were significantly increased after 8 weeks of HFD (*p* < 0.001), while the level of high-density lipoprotein cholesterol (HDL-C) was significantly decreased (*p* < 0.001) (Figure 7A–F). The level of TG showed an upward trend. However, the treatment with PCELNs significantly decreased the levels of TC, TG, ALT, and AST (*p* < 0.05, *p* < 0.01, *p* < 0.001), while it significantly increased the level of HDL-C (*p* < 0.001). These results indicated that PCELNs regulated HFD-induced dyslipidemia.

#### 2.6.3. Effects of PCELNs on Liver Lipid Metabolism

**Figure 8:**
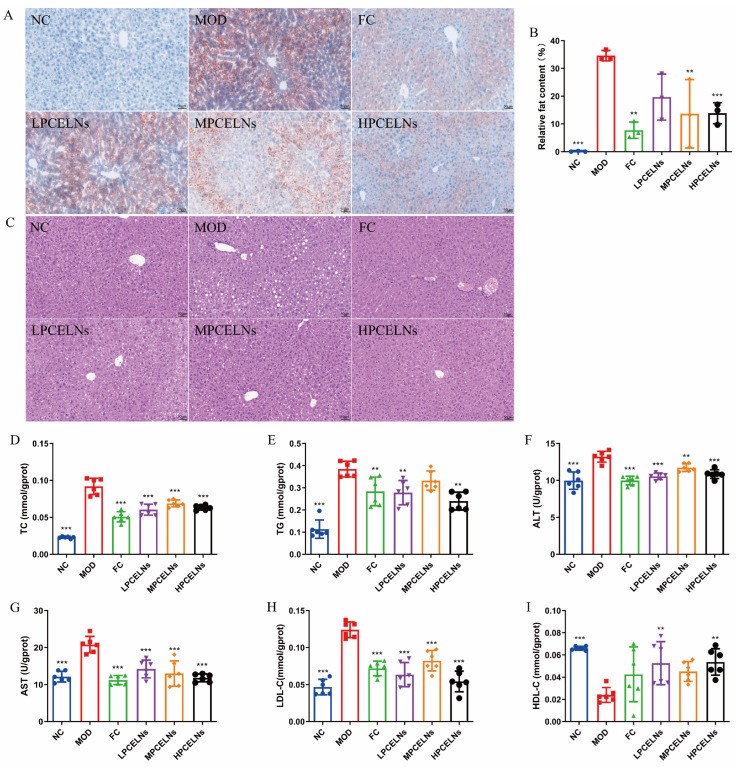
Effect of PCELNs on liver lipid metabolism. (**A**) Images of oil red O staining (scale bar = 50 µm). (**B**) Analysis of relative fat content. (**C**) Hematoxylin and eosin (H&E) images. Liver levels of (**D**) TC, (**E**) TG, (**F**) ALT, (**G**) AST, (**H**) LDL-C, and (**I**) HDL-C. *n* = 6. Results were expressed as mean ± S.D. ** *p* < 0.01, *** *p* < 0.001 vs. MOD group.

Abnormal lipid metabolism in the liver is involved in the development of MAFLD. Mouse liver showed a severe lipid accumulation, as well as diffuse hepatocellular edema and degeneration after 8 weeks of HFD (Figure 8A–C). The treatment with PCELNs reduced the swelling and degeneration of hepatocytes, and the pathological morphology of liver returned to the normal state. Similarly, Figure 8D–I shows that the levels of TC, TG, ALT, AST and LDL-C significantly increased (*p* < 0.001), while the level of HDL-C was significantly decreased (*p* < 0.001) after 8 weeks of HFD. The treatment with PCELNs significantly decreased the levels of TC, TG, ALT, AST, and LDL-C (*p* < 0.01, *p* < 0.001), while significantly increased the level of HDL-C (*p* < 0.01). These results suggested that PCELNs regulated HFD-induced liver lipid disorder.

#### 2.6.4. Effects of PCELNs on Serum and Liver Inflammatory Factors

**Figure 9:**
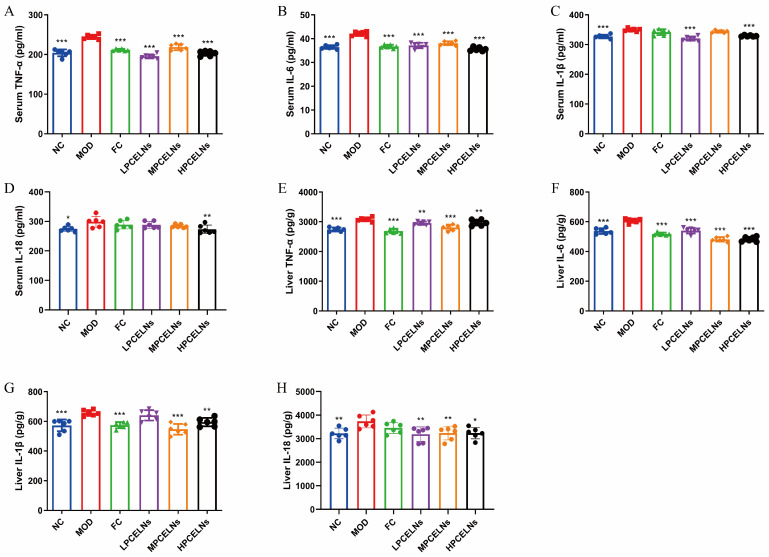
Effect of PCELNs on serum and liver inflammatory factors. Serum levels of (**A**) TNF-α, (**B**) IL-6, (**C**) IL-1β, and (**D**) IL-18. Expression of (**E**) TNF-α, (**F**) IL-6, (**G**) IL-1β, and (**H**) IL-18 in liver. *n* = 6. Results were expressed as mean ± S.D. * *p* < 0.05, ** *p* < 0.01, *** *p* < 0.001 vs. MOD group. TNF-α, tumor necrosis factor-α; IL-6, interleukin-6; IL-1β, interleukin-1β; IL-18, interleukin-18.

Eight weeks of HFD significantly increased the levels of tumor necrosis factor-α (TNF-α), interleukin-6 (IL-6), interleukin-1β (IL-1β), and interleukin-18 (IL-18) in the serum (*p* < 0.05 and *p* < 0.001), as well as their expression in the liver (*p* < 0.01 and *p* < 0.001) (Figure 9A–H). The treatment with PCELNs significantly decreased the levels of TNF-α, IL-6, IL-1β, and IL-18 in the serum (*p* < 0.01, *p* < 0.001), as well as their expression in the liver (*p* < 0.05, *p* < 0.01, *p* < 0.001). These results indicated that PCELNs inhibited the inflammatory response to alleviate MAFLD.

#### 2.6.5. Effects of PCELNs on Mitochondrial Ultrastructure and Function in Liver

**Figure 10:**
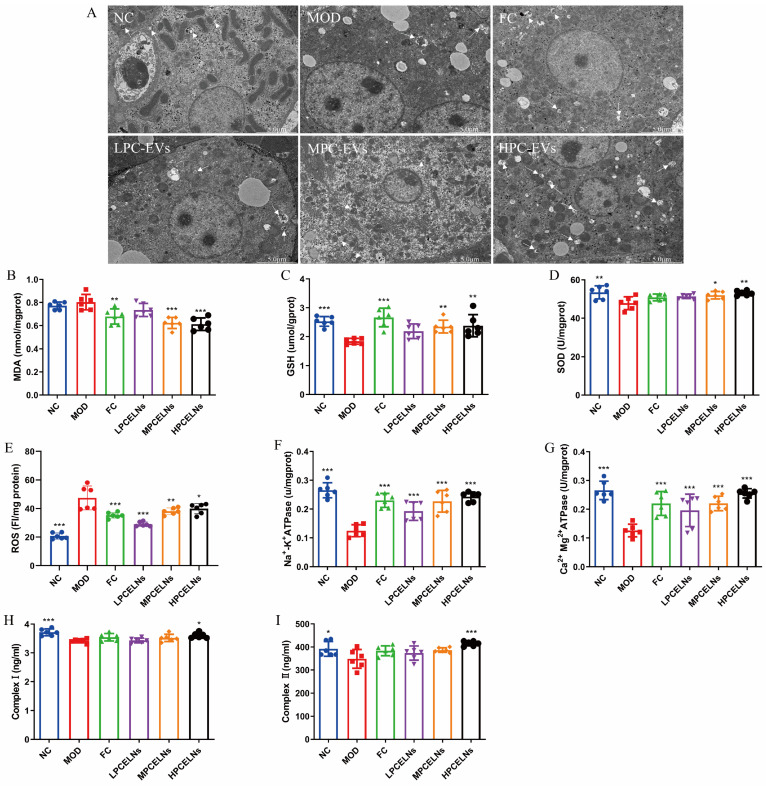
Effect of PCELNs on mitochondrial ultrastructure and function in liver. (**A**) TEM image of mitochondria (scale bar = 5 µm). Levels of (**B**) MDA, (**C**) GSH, (**D**) SOD, (**E**) ROS, (**F**) Na^+^-K^+^-ATPase, (**G**) Ca^2+^-Mg^2+^-ATPase, (**H**) complex I, and (**I**) complex II. *n* = 6. Results were expressed as mean ± S.D. * *p* < 0.05, ** *p* < 0.01, *** *p* < 0.001 vs. MOD group. MDA, malondialdehyde.

The ultrastructure of liver mitochondria showed swelling and degeneration, the inner and outer membranes and cristae of mitochondria were blurred, and a few autophagosomes appeared after 8 weeks of HFD (Figure 10A, white arrow). However, PCELN treatment significantly improved the mitochondrial ultrastructure and the number of autophagosomes in the liver. Subsequently, the relevant indicators of mitochondrial function revealed that 8 weeks of HFD significantly increased the ROS level (*p* < 0.001), and significantly decreased the activities of SOD, GSH, Na^+^-K^+^-ATPase, Ca^2+^-Mg^2+^-ATPase, and complex I and II (*p* < 0.05, *p* < 0.01, *p* < 0.001) (Figure 10B–I). After treatment with PCELNs, the activities of SOD, GSH, Na^+^-K^+^-ATPase, Ca^2+^-Mg^2+^-ATPase, and complex I and II were significantly increased (*p* < 0.05, *p* < 0.01, *p* < 0.001), while the levels of malondialdehyde (MDA) and ROS were significantly decreased (*p* < 0.05, *p* < 0.01, *p* < 0.001). The above results indicated that PCELNs improved mitochondrial ultrastructure and regulated mitochondrial function (oxidative stress and energy metabolism) to alleviate MAFLD.

#### 2.6.6. Mitophagy Was Involved in the Inhibition of NLRP3 Inflammasome by PCELNs

**Figure 11:**
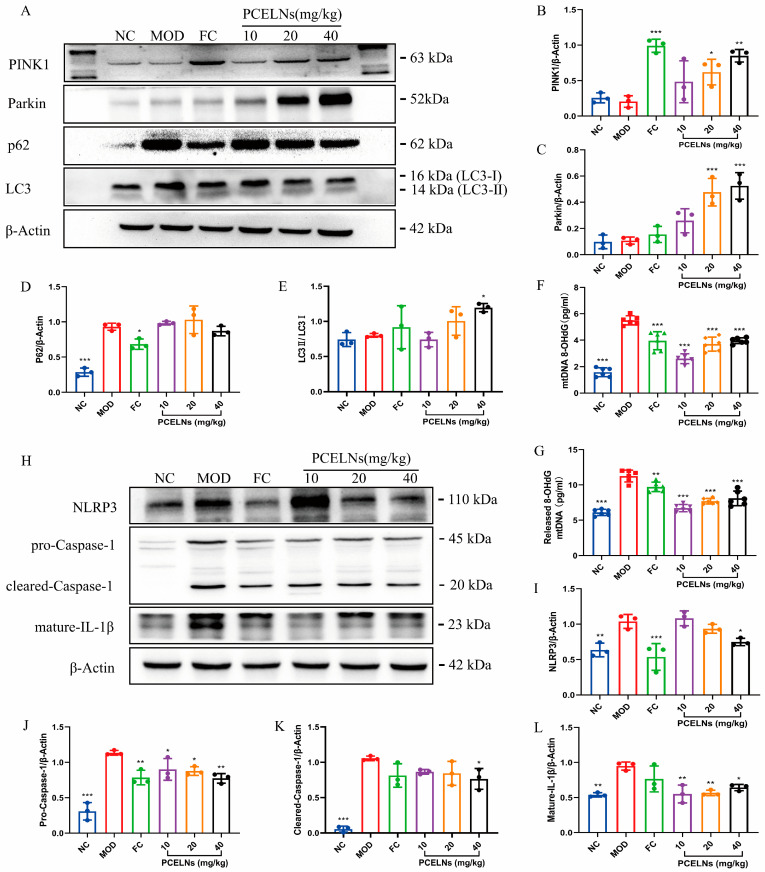
Mitophagy was involved in inhibition of NLRP3 inflammasome by PCELNs. (**A**) Immunoblotting images of PINK1, Parkin, p62, and LC3. Relative expression of (**B**) PINK1, (**C**) Parkin, (**D**) p62, and (**E**) LC3. Levels of (**F**) mitochondrial Ox-mtDNA and (**G**) cytoplasmic Ox-mtDNA. (**H**) immunoblotting images of NLRP3, pro-caspase-1, cleared-caspase-1, and mature-IL-1β. Relative expression of (**I**) NLRP3, (**J**) pro-caspase-1, (**K**) cleared-caspase-1, and (**L**) mature-IL-1β. *n* = 3. Results were expressed as mean ± S.D. * *p* < 0.05, ** *p* < 0.01, *** *p* < 0.001 vs. MOD group. NLRP3, nucleotide binding oligomerization domain-like receptor protein 3; caspase-1, cysteinyl aspartate specific proteinase-1; Ox-mtDNA, oxidized mitochondrial DNA; 8-OHdG, 8-hydroxy-desoxyguanosine.

The occurrence and development of MAFLD are closely related to mitophagy and oxidative damage of mitochondrial DNA. Therefore, the expression of mitophagy-related proteins, the Ox-mtDNA level, and the expression of NLRP3 inflammasome-related proteins were measured. p62 protein expression was significantly increased (*p* < 0.001), while PINK1 protein expression showed a reduced trend after 8 weeks of HFD (Figure 11A–E). However, the treatment with PCELNs significantly increased PINK1, Parkin, and LC3 protein expression (*p* < 0.001), while p62 protein expression showed a reduced trend. Mitochondrial and cytosolic Ox-mtDNA levels were significantly increased (*p* < 0.001), while the treatment with PCELNs significantly reversed this change after 8 weeks of HFD (*p* < 0.001) (Figure 11F,G). NLRP3, pro-cysteinyl aspartate specific proteinase-1 (caspase-1), cleared-caspase-1, and mature-IL-1β protein expression were significantly increased after 8 weeks of HFD (*p* < 0.01, *p* < 0.001) (Figure 11H–L). However, treatment with PCELNs significantly decreased their expression (*p* < 0.05, *p* < 0.01). The above results confirmed that PCELNs up-regulated PINK1/parkin-mediated mitophagy and reduced ROS-induced mitochondrial DNA oxidative damage, which further inhibited NLRP3 inflammasome activation to improve MAFLD.

## 3. Discussion

In recent years, the components of ELNs have been revealed, such as proteins, lipids, nucleic acids, and homologous secondary metabolites [16]. In addition, ELNs have a wide range of sources, low toxicity, unique physicochemical properties, and targeted delivery, which make them a promising candidate in the treatment of human diseases [16]. This study described the isolation of new HELNs from PC for the first time and investigated their characteristics and biological activity. The results confirmed that the morphology, particle size, and zeta potential of PCELNs were similar to the those of ELNs reported in the literature [24]. In addition, the analysis of the composition of PCELNs revealed the presence of various bioactive substances, including lipids, proteins, and nucleic acids, which were similar to those of ELNs reported in the literature [24]. The presence of homologous active small molecule compounds in ELNs was reported [15], but the active small molecules (triterpenoids) in PC from PCELNs have not yet been discovered by ultra-high performance liquid chromatography–mass spectrometry (UHPLC/MS). The lipid composition of PCELNs showed that TG content was the highest (more than 30%), followed by Cer, PE, DG, and DGMG. Glycerophospholipids are the main phospholipid components of the cell membrane, and they contribute to the maintenance of the membrane integrity and stability of HELNs [25]. Cer is involved in cell signal transduction and may participate in the regulation of the formation and release of HELNs, affecting the exchange of information and material transfer among cells [24]. Furthermore, our results revealed multiple miRNAs in PCELNs, and gra-miR-8705 and osa-miR-2275c had the highest abundance among the top 10 miRNAs. Finally, the target proteins of the top 10 miRNAs were analyzed and were related to the regulation of pathways in cancer and metabolic pathways. ELNs, including PCELNs, have different characteristics compared to lipid nanoparticles (LNPs). ELNs present a cuplike morphology, whereas LNPs are spherical or quasi-spherical. The particle size of ELNs ranges from 100 to 200 nm, while the particle size of LNPs can be adjusted according to the substances they encapsulate. ELNs also feature low immunogenicity, good biocompatibility, and strong targeting capabilities. Additionally, ELNs can also be used as drug carriers (such as vaccines), which is similar to LNPs. Currently, there have already been studies on ELNs as carriers for cancer vaccines in both clinical and basic research [26,27,28]. Therefore, PCELNs have the potential to be developed into drug carriers.

MAFLD currently affects nearly a quarter of the world’s population and has become a serious public health problem worldwide [29]. It is a disease characterized by an excessive accumulation of lipids in the liver due to metabolic disorders [30]. The pathophysiology of MAFLD involves excessive liver fat accumulation, hepatocyte dysfunction, inflammation, and fibrosis, with subsequent risk of hepatocellular carcinoma [31]. This study revealed that PCELNs were absorbed by L02 cells as well as by the liver and spleen of mice. In addition, the steatosis model of L02 cells induced by fat emulsion and MAFLD mouse model induced by 8 weeks HFD were used to evaluate the therapeutic effects of PCELNs and their potential mechanism, with fenofibrate capsules (FC) as positive control (FC is frequently used as a positive control for the study of drugs against MAFLD [32,33,34]). The occurrence of MAFLD is related to an excessive accumulation of lipids in the liver (exceeding its own clearance capacity), which is characterized by increased TC, TG, AST, ALT, and LDL-C content and decreased HDL-C content in the serum and liver [35]. The findings in the present cell and animal experiments indicated that PCELNs not only reversed these biochemical indexes, but also reduced the body weight, liver index, spleen index, WATg index, and BAT index of mice, thus improving HFD-induced MAFLD. Furthermore, histomorphology and pathological results showed that HFD induced an evident accumulation of lipid droplets in the liver, while PCELNs decreased them, and the pathological morphology of the liver returned to normal. In addition, the effects of the three doses of PCELNs on the TC, TG, and ALT levels in L02 cells exhibited a non-linear dose–response. This may be attributed to the complex composition of PCELNs and the underlying pharmacological mechanisms (for example, the mode of action featuring multiple components, multiple pathways, and multiple targets), or the dosages used might not fall within the range of the dose–response of PCELNs.

Mitochondrial dysfunction is closely related to the development of MAFLD. Studies showed the occurrence of mitochondrial dysfunction and oxidative stress in the liver of MAFLD patients, which are manifested as mitochondrial ultrastructural damage, decreased respiratory chain activity, and excessive production of ROS [36]. In this study, fat emulsion and HFD significantly promoted oxidative stress (indicated by SOD, GSH, MDA, and ROS levels) and energy metabolism disorder (indicated by ATPase, as well as complex I and II levels) in liver mitochondria, and the swelling and degeneration of mitochondrial ultrastructure, which accelerated the development of MAFLD. These changes were effectively reversed by PCELNs, indicating their ability to improve mitochondrial oxidative stress, energy metabolism, and ultrastructure.

**Figure 12:**
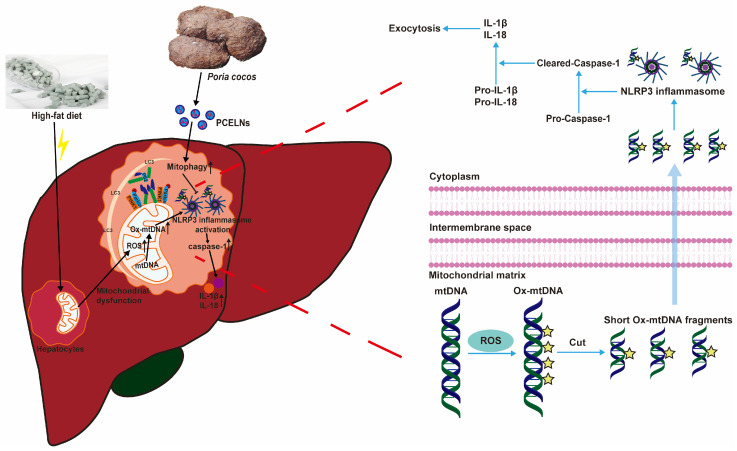
PCELNs alleviated HFD-induced MAFLD by promoting mitophagy and inhibiting NLRP3 inflammasome activation.

Excessive lipid accumulation in MAFLD inhibits mitophagy, thereby promoting the inflammatory response [37]. Mitophagy is a key player in MAFLD, which removes damaged mitochondria and prevents the progression of MAFLD caused by oxidative stress [36]. In addition, the excessive production of ROS, which leads to mtDNA damage, promotes NLRP3 inflammasome-mediated inflammatory response, and aggravates the development of MAFLD [36,38]. PINK1/Parkin-mediated mitophagy, which is a classical mitophagy pathway, helps to improve mitochondrial dysfunction, reduce ROS production, and balance metabolic homeostasis, thereby protecting or preventing the development of MAFLD [39]. In the present study, PCELNs were able to modulate fat emulsion and HFD-induced mitophagy inhibition (indicated by PINK1, Parkin, LC3, and p62 levels) to further alleviate MAFLD. However, this effect was reversed by PCELNs, indicating that they regulated mitophagy and improved mitochondrial dysfunction to further alleviate MAFLD. Subsequently, HFD induced the activation of NLRP3 inflammasome (indicated by Ox-mtDNA, NLRP3, caspase-1, and IL-1β levels). The effect was reversed by the treatment with PCELNs, indicating that they reduced Ox-mtDNA production and efflux and inhibited NLRP3 inflammasome activation to further alleviate MAFLD (Figure 12).

ELNs have been used in clinical practice. The latest clinical treatment has shown that rose stem cell-derived ELNs for the treatment of androgenetic alopecia accompanied by poliosis not only lead to hair regeneration but also result in the repigmentation of gray hair [40]. In the study, almost all MAFLD-related biomarkers (e.g., TC, TG, ALT, AST, HDL-C, LDL-C) were restored to normal status after the intervention with PCELNs, showing statistical significance (*p*-values). These results sufficiently indicate that PCELNs are a potential drug for clinically treating MAFLD. Although the yield of PCELNs extracted from PC using ultracentrifugation was 0.74%, PCELNs can be produced in large quantities for clinical use through plant cell culture technology. The feasibility of plant cell culture technology for the large-scale production of ELNs was proved [21].

## 4. Materials and Methods

### 4.1. Chemicals, Reagents, and Materials

HFD (40% fat, 20% fructose, and 2% cholesterol) was purchased from SYSE Biotech. Co., Ltd. (Changzhou, China). Basal feed was purchased from SiPeiFu Biotech. Co., Ltd. (Beijing, China). RPMI-1640 culture medium, penicillin/streptomycin, and trypsin were purchased from Gibco (Waltham, MA, USA). Fetal bovine serum was purchased from Procell Life Science & Technology Co., Ltd. (Wuhan, China). Phosphate-buffered saline (PBS) was purchased from Saiguo Technology Co., Ltd. (Guangzhou, China). Cell Counting Kit-8 was purchased from Bioss Biotech Co., Ltd. (Beijing, China). FC were purchased from Abbott Laboratories (Abbott Park, IL, USA). Fat emulsion was purchased from the Yunnan Provincial Hospital of Traditional Chinese Medicine (Kunming, China). Kits to measure TG, TC, ALT, AST, LDL-C, HDL-C, MDA, SOD, GSH, and ATPase were purchased from Jiancheng Bioengineering Institute (Nanjing, China). Kits to measure TNF-α, IL-6, IL-1β, IL-18, and complex I and II were purchased from Jiangsu Meimian Industrial Co., Ltd. (Yancheng, China). ROS kit and DIR (near-infrared fluorescence probe for cell membrane) were purchased from Suzhou UElandy Biotech Co., Ltd. (Suzhou, China). PKH26 red fluorescent cell linker mini kit and DAPI were purchased from Sigma-Aldrich (St. Louis, MO, USA). The 8-Hydroxy-desoxyguanosine (8-OHdG) kit was purchased from Wuhan Cusabio Co., Ltd. (Wuhan, China). Additionally, 4% paraformaldehyde and electron microscope fixative were purchased from Servicebio Biotech Co., Ltd. (Wuhan, China). The hematoxylin and eosin (H&E) stain kit, oil red O stain kit, RIPA lysis buffer, and 5× loading buffer were purchased from Solarbio Co., Ltd. (Shanghai, China). The mitochondrial isolation kit and bicinchoninic acid (BCA) protein assay kit were purchased from Shanghai Beyotime Biotech Co., Ltd. (Shanghai, China). Anti-PINK1 (Cat No. 23274-1-AP), anti-p62 (Cat No. 18420-1-AP), and anti-NLRP3 (Cat No. 27458-1-AP) were purchased from Proteintech (Wuhan, China). Anti-Parkin (Cat No. ab77924) was purchased from Abcam (Cambridge, UK). Anti-LC3 (Cat No. 4108) was purchased from Cell Signal Technology (Danvers, MA, USA). Anti-IL-1β (Cat No. sc-12742) was purchased from Santa Cruz Biotechnology (Dallas, TX, USA). Anti-caspase-1 (Cat No. A16288) was purchased from ABclonal (Woburn, MA, USA). The Omni-ECL™ Pico Light Chemiluminescence Kit was purchased from Shanghai Epizyme Biotech Co., Ltd. (Shanghai, China). The Blue Plus^®^ IV Protein Marker and EasyPure^®^ Genomic DNA kit were purchased from TransGen Biotech Co., Ltd. (Beijing, China). All the other reagents were of analytical grade or higher. The PC samples were purchased from Medicinal Herb Market in Pu’er City (China) and authenticated by Professor Jie Yu at the Yunnan University of Chinese Medicine, and stored in the Key Laboratory of Preventing Metabolic Diseases of traditional Chinese medicine, Yunnan University of Chinese Medicine (Kunming, China).

### 4.2. Preparation and Characterization of PCELNs

Fresh PC samples were peeled, cut into pieces, and washed thoroughly with purified water. Subsequently, the PC samples were homogenized in PBS using a blender. The PC juice was centrifuged at 1000× *g* for 10 min, 3000× *g* for 20 min, and 10,000× *g* for 30 min at 4 °C, and filtered using a 0.22 um filter membrane to remove the insoluble residue. The supernatant was centrifuged at 150,000× *g* for 2 h at 4 °C. The precipitate containing PCELNs was immediately collected and stored at −80 °C until use.

DLS (NanoBrook 90Plus, Brookhaven Instruments, Nashua, NH, USA) and NTA (ZetaView, Particle Metrix, Meerbusch, NRW, Germany) were used to measure the size and zeta potential of PCELNs. PCELNs’ morphology was observed and photographed using TEM (HT-7700, Hitachi, Tokyo, Japan), as previously described [41].

PCELNs were lysed using RIPA lysis buffer for 10 min on an ice bath and centrifuged at 12,000× *g* for 5 min at 4 °C. Six microliter of the supernatant containing proteins were separated by 10% SDS-PAGE, stained with Coomassie blue, and decolorized to observe the distribution of the proteins.

Total lipids were extracted using the Folch method [42]. A mixture of chloroform/methanol (2:1) was added to the suspension of PCELNs. The organic phase was collected, shaken dry, and subsequently resuspended in chloroform. The lipid distribution was observed on silica gel thin-layer chromatography (TLC) plates using chloroform/methanol/glacial acetic acid (190:9:1) as the development agent and visualized using a 10% CuSO_4_ and 8% phosphoric acid reagent. Lipid samples (1 mL) from PCELNs were submitted to Suzhou PANOMIX Biomedical Tech Co., Ltd. (Suzhou, China) to perform lipidomic analysis. The lipid composition of PCELNs was detected using liquid chromatography (Vanquish, Thermo Fisher Scientific, Shanghai, China) and mass spectrometer (Q Exactive, Thermo Fisher Scientific, Shanghai, China). Subsequently, data were processed and analyzed using LipidSearch software v.4.2.23 (Thermo Fisher Scientific, Shanghai, China).

The miRNA-seq library was constructed using QIAseq^®^ miRNA Library Kit (Qiagen, Germantown, MD, USA), and high throughput sequencing of miRNAs was performed by Beijing Qinglian Biotech Co., Ltd. (Beijing, China) on the Illumina NovaSeq6000 platform (San Diego, CA, USA), which used a double-end sequencing strategy to sequence millions of miRNAs at a time. The miRNAs in PCELNs were confirmed by aligning the sequences of known miRNAs in the official database with the tested miRNAs. Subsequently, the target genes of the candidate miRNAs were predicted using the algorithms of the databases miRanda (http://www.microrna.org/microrna/home.do, accessed on 6 October 2024) and TargetScan (https://www.targetscan.org/vert_72/, accessed on 6 October 2024). Finally, target genes were analyzed using Gene Ontology (GO) (https://geneontology.org/, accessed on 8 October 2024) and the Kyoto Encyclopedia of Genes and Genomes (KEGG) (https://www.genome.jp/kegg, accessed on 8 October 2024) pathway and visualized using the Database for Annotation, Visualization, and Integrated Discovery (DAVID) (https://davidbioinformatics.nih.gov/, accessed on 9 October 2024), as well as SRplot data visualization processing platforms (http://www.bioinformatics.com.cn/SRplot, accessed on 9 October 2024).

The active small molecular compounds in PCELNs were assessed using an Agilent 1290 Infinity II Ultra-high Performance Liquid Chromatography plus Agilent G6545 Quadrupole Time-of-Flight Mass Spectrometer (Agilent Technologies, Santa Clara, CA, USA) coupled with a ACQUITY UPLC^®^ HSS T3 (2.1 × 100 mm, 1.8 µm) column (Waters, Milford, MA, USA). Methanol was added to PCELNs. After thorough mixing, the mixture was placed in liquid nitrogen for 5 min. Subsequently, it was transferred to an ice bath and allowed to stand for 3 min. This freeze–thaw cycle was repeated three times. Then, the sample was sonicated for 3 min and then centrifuged at 10,000× *g* for 10 min. The supernatant was collected and reserved for analysis. The analytical conditions of UHPLC were the following: the mobile phase was composed of 0.1% formic acid water (A) and acetonitrile (B) with a gradient program (0–50 min, 5–90% B; 50–55 min, 90% B; 55–56 min, 90–5% B; 56–60 min, 5% B). The flow rate was 0.2 mL/min, the detection wavelength was 250 nm, the column temperature was 30 °C, and the sample injection volume was 5 μL. The MS was operated under positive and negative ion mode, and ESI was the ion source. The capillary temperature and heat block temperature were set at 350 °C and 250 °C, respectively. The nebulizing nitrogen gas flow was 1.5 L/min, with the interface voltage (+) 3.5 kV, (−) −2.8 kV, mass range MS, *m*/*z* 100–1200 and MS^2^, *m*/*z* 50–1000. The Mass Hunter Qualitative Analysis B.06.00 was used to perform the small molecules in PCELNs by comparison with previously reported MS and MS*^n^* data.

### 4.3. Cell Experiments

#### 4.3.1. Cell Culture

Human hepatocyte cell line L02 (iCell-h054) was purchased from Cellverse Bioscience Technology Co., Ltd. (Shanghai, China). L02 cells were cultured in RPMI-1640 medium supplemented with 10% fetal bovine serum and 1% penicillin-streptomycin and incubated in a humid environment at 37 °C and 5% CO_2_. The cells were digested with 0.25% trypsin, counted, and then seeded into a 6-well plate (3 × 10^5^ cells/well).

#### 4.3.2. Labeling and Uptake of PCELNs

The PCELNs’ precipitate and the PKH26 staining solution were, respectively, dissolved in diluent C according to the instructions provided by the kit. Subsequently, the two solutions were mixed, incubated at room temperature for 5 min, and finally quenched by adding 10% BSA solution. PKH26-PCELNs were precipitated by centrifugation at 150,000× *g* for 2 h, resuspended in PBS, and incubated with L02 cells for 1, 3, 6, 12, and 24 h. The nuclei were stained with DAPI. The cells were washed 3 times with PBS and fixed with 4% paraformaldehyde. Images were acquired using an inverted fluorescence microscope imaging system (Ti-S, Nikon, Tokyo, Japan).

#### 4.3.3. Cytotoxicity Assay

The cytotoxicity of PCELNs on L02 cells was evaluated by MTT assay. PCELNs (10–100 µg/mL) were incubated with L02 cells for 24 and 48 h, then cell viability was measured using Cell Counting Kit-8.

#### 4.3.4. Therapeutic Effect of PCELNs on Steatosis of L02 Cells

L02 cells were seeded into a 6-well plate until reaching 80 to 90% confluence. Then, they were cultured in medium containing 0.2% serum for 12 h. Subsequently, the cells were cultured in medium containing 5% medical fat emulsion for 24 h to induce steatosis. Next, the cells were treated with FC (150 µmol/L) and PCELNs (10, 50 and 100 µg/mL) for 24 h, then collected and the content of TG, TC, AST, ALT, SOD, GSH, ATPase, and complex I and II was detected according to the operating instructions of the kit. Absorbance was read on a microplate reader (SpectraMax Plus 384, Molecular Devices, San Jose, CA, USA).

Lipid accumulation was evaluated by oil red O staining. The cells were fixed with 4% paraformaldehyde for 30 min, followed by the addition of 60% isopropanol and incubation for 1 min. Oil red O staining solution was added, the cells were incubated for 20 min, then washed with distilled water to remove residual staining solution and counterstained with hematoxylin for 2 min. Finally, the cells were washed again with distilled water, and images were taken using an imaging system (CX31, Olympus Corporation, Tokyo, Japan).

Cellular ROS level was detected using flow cytometry. L02 cells were seeded into a 6-well plate (1 × 10^5^ cells/well) and incubated for 24 h. Next, the cells were cultured in medium containing 0.2% serum for 12 h and in medium containing 5% medical fat emulsion for 24 h. Subsequently, they were treated with FC and PCELNs for 24 h and washed with PBS. The DCFH-DA probe was added, and the cells were incubated in the dark for 30 min. Next, the cells were digested with 0.25% trypsin, and the cell suspension was collected and centrifuged at 1000 rpm for 3 min at room temperature. Cell precipitation was resuspended in PBS and ROS were detected by flow cytometer (FACSCelesta, Becton, Dickinson and Company, Franklin Lakes, NJ, USA).

PINK1, Parkin, p62, and LC3 protein expression were measured by immunofluorescence. L02 cells were seeded on circular cover glass and cultured into a 6-well plate for 24 h. Cell model was constructed and treated as described above. Cell slides were then fixed, permeabilized, and incubated with primary antibodies (PINK1, Parkin, p62, and LC3) at 4 °C overnight, followed by staining with secondary fluorescent antibodies Alexa Fluor 488 (Servicebio Biotech Co., Ltd., Wuhan, China) at room temperature for 1 h and counterstained with DAPI for 10 min. Finally, images were taken using a confocal microscope (Eclipse C1 Plus, Nikon, Tokyo, Japan).

### 4.4. Animal Experiments

#### 4.4.1. Animals

Healthy C57BL/6 male mice (8 weeks old; 20 ± 2 g; quality certificate number: SCXK(Jing)2019-0010) were provided by SiPeiFu Biotech. Co., Ltd. (Beijing, China). The mice were maintained in a pathogen-free environment, under 12 h light/dark cycles, at a temperature of 23 ± 2 °C, and 60% relative humidity; standard food and water were provided ad libitum. 

#### 4.4.2. Imaging In Vivo and Organ Distribution of PCELNs

The biodistribution of PCELNs was explored by incubating them with DIR staining solution for 30 min at 37 °C according to the instructions provided by the kit. DIR-PCELNs were precipitated by centrifugation at 150,000× *g* for 2 h and resuspended in PBS. Seven C57BL/6 mice were fasted for 12 h and depilated in advance. Six mice were treated with a single dose of 40 mg/kg DIR-PCELNs by intraperitoneal injection (0.1 mL/10 g body weight), and one was treated with a single dose of 40 mg/kg PCELNs by intraperitoneal injection (0.1 mL/10 g body weight). Subsequently, the distribution of DIR-PCELNs in vivo was detected by an animal in vivo imaging system (IVIS Lumina Series III, PerkinElmer, Waltham, MA, USA). The mice were dissected at the indicated times, and the heart, liver, spleen, lung, kidney, and brain were collected for fluorescence imaging after washing them with saline.

#### 4.4.3. Therapeutic Effect of PCELNs Against MAFLD

##### Design

C57BL/6 mice were adaptively fed for one week. Subsequently, they were randomly divided into six groups (six mice in each group): (1) normal control group (NC; normal saline); (2) model group (MOD; HFD and normal saline); (3) FC group (HFD and FC 20 mg/kg/d); (4) low PCELN-treated group (HFD and LPCELNs 10 mg/kg/d); (5) middle PCELN-treated group (HFD and MPCELNs 20 mg/kg/d); and (6) high PCELN-treated group (HFD and HPCELNs 40 mg/kg/d). The mice were treated with an intraperitoneal injection (0.1 mL/10 g body weight) once a day for 8 weeks. The mice were fed with HFD (40% fat, 20% fructose, and 2% cholesterol) for 8 weeks to induce MAFLD. Mouse weight was recorded once a week during the experiment, and food intake was recorded once a day.

##### Sample Collection

The mice were anesthetized with 1% sodium pentobarbital intraperitoneally injected at a dose of 0.05–0.1 mL/10 g body weight after the last administration at week 8. Blood was collected from the abdominal aorta, centrifuged for 15 min (4 °C; 3500 rpm), and the serum was collected, which was stored at −80 °C until analysis. Heart, liver, spleen, lung, kidney, inguinal WATi, WATg, and BAT were collected and weighed. A portion of liver was stored in 4% paraformaldehyde and electron microscope fixative. The remaining liver and other tissues were stored at −80 °C for subsequent analysis.

##### Histopathological Assessment

Histopathological assessment was performed by H&E staining. In brief, samples of liver taken from the same location and embedded in paraffin were fixed, cut into 4 µm thick sections using a freezing microtome (CryoStar NX50, Thermo Fisher Scientific, Shanghai, China), and stained with H&E. Subsequently, the sections were sealed with neutral gum and images were acquired using an imaging system (CX31, Olympus Corporation, Tokyo, Japan).

Oil red O staining was performed in samples of liver taken from the same location, embedded in plastic embedding boxes, and frozen at −20 °C for 30 min. Tissues were cut into 8 µm thick sections using a freezing microtome, fixed on slides, and immersed in 60% isopropanol for 5 s. The sections were stained with oil red O for 15 min, washed with PBS, and counterstained with hematoxylin for 10 s. Subsequently, the sections were sealed with glycerin gelatin and images were acquired using an imaging system (CX31, Olympus Corporation, Tokyo, Japan).

##### TEM of the Liver

Liver samples were fixed overnight in 2.5% glutaraldehyde and post-fixed in 1% osmium tetroxide. The samples were dehydrated with ethanol and acetone (20%, 30%, 50%, 70%, 90%, and 100%), embedded in Epon resin, cut by a microtome (UC6, Leica, Wetzlar, Germany), and stained with uranyl acetate and lead citrate. The images were collected using TEM (JEM-1400, JEOL, Tokyo, Japan).

##### Serum and Liver Biochemical Parameters

TC, TG, ALT, AST, HDL-C, and LDL-C content in the serum was determined by a biochemical analyzer (CX4, Beckman, Krefeld, Germany). Their content in the liver was determined according to the kit manufacturer’s instructions, and absorbance was read on a microplate reader. The content of inflammation factors in the serum and liver, including TNF-α, IL-6, IL-1β, and IL-18, was also determined according to the kit manufacturer’s instructions, and absorbance was read on a microplate reader.

##### Oxidative Stress and Energy Metabolism in the Liver

SOD, MDA, GSH, ATPase, and complex I and II levels in the liver were determined according to the kit manufacturer’s instructions, and absorbance was read on a microplate reader. ROS level was determined by DCFH-DA assay [43]. Briefly, the sample was treated with 10 μM DCFH-DA and incubated at 37 °C for 30 min in the dark. Fluorescence intensity was read using a fluorescence microplate reader (SuPerMax 3000FA, Flash Biotechnology Co., Ltd., Shanghai, China).

##### Ox-mtDNA in Liver Mitochondria and Cytoplasm

Liver samples were cut into pieces, normal saline in a 10 times larger volume was added, and they were homogenized on ice for approximately 15 times. The homogenized tissue was centrifuged at 1000× *g* and 4 °C for 5 min. The supernatant was further centrifuged at 11,000× *g* and 4 °C for 10 min to separate the cytosolic and mitochondrial parts (the supernatant contained the cytoplasm, and the precipitation contained the crude mitochondria). Mitochondria were purified from the precipitate according to the instructions of the mitochondrial isolation kit. Ox-mtDNA was measured by purifying DNA from the mitochondrial and cytosolic fractions using the EasyPure^®^ Genomic DNA kit. Then, 8-OHdG content in the cytoplasm and mitochondria was measured according to the instructions of the 8-OHdG kit, and absorbance was read on a microplate reader.

##### Western Blot Analysis

The liver was cut into pieces and homogenized in RIPA lysis buffer supplemented with PMSF (protease inhibitor) for 30 min, and total proteins were obtained by centrifugation at 12,000 rpm and 4 °C for 10 min. Subsequently, the protein concentration was quantified using the BCA protein assay kit. Moreover, 5× Sample Buffer was added to all protein samples that were boiled for 5 min. The proteins were separated by 10% or 12% sulfate-polyacrylamide gel electrophoresis and transferred to a polyvinylidene fluoride membrane. The membrane was blocked with 5% skim milk for 2 h at room temperature and then incubated at 4 °C overnight with the following primary antibodies: PINK1 (1:2000), Parkin (1:2000), LC3 (1:2000), p62 (1:5000), NLRP3 (1:5000), IL-1β (1:1000), caspase-1 (1:4000), and β-actin (1:4000). Subsequently, the membrane was washed and incubated with goat anti-mouse IgG (1:5000) and goat anti-rabbit IgG (1:5000) for 1 h at room temperature, then washed. Enhanced chemiluminescence solution was used to react with the membrane, and the gray bands were observed in the chemiluminescence imaging analysis system (Tanon 5200, Tanon, Shanghai, China).

### 4.5. Statistical Analysis

Statistical analysis was performed using GraphPad Prism 9.0 (GraphPad Software, La Jolla, CA, USA). The results were expressed as mean ± standard deviation. One-way analysis of variance (ANOVA, Dunnett method) was used to assess the difference among the groups. A value of *p* < 0.05 was considered statistically significant.

## 5. Conclusions

PCELNs alleviated HFD-induced MAFLD and multiple risk factors by restoring mitochondrial function, including the protection of mitochondrial ultrastructure, alleviation of oxidative stress damage, and improvement of energy metabolism, which were related to the promotion of PINK1/Parkin-mediated mitophagy, the reduction in Ox-mtDNA production, as well as efflux and inhibition of NLRP3 inflammasome activation. Therefore, PCELNs might be a potential therapeutic agent for the prevention and treatment of MAFLD, and may also provide a new strategy for discovering new drug candidates among herbal medicines.

## Data Availability

All data used to support the findings of this study are available from the corresponding author upon reasonable request.

## Abbreviations

ALT, alanine transaminase; AST, aspartate transaminase; ATPase, ATP synthase; BAT, brown adipose tissue; BCA, bicinchoninic acid; caspase-1, cysteinyl aspartate specific proteinase-1; Cer, ceramide; complex I, respiratory chain complex I; complex II, respiratory chain complex II; DAVID, Database for Annotation, Visualization, and Integrated Discovery; DG, diacylglycerol; DGMG, digalactosyl monoacylglycerol; DLS, dynamic light scattering; ELNs, exosome-like nanovesicles; FC, fenofibrate capsules; GO, Gene Ontology; GSH, glutathione; H&E, hematoxylin and eosin; HDL-C, high-density lipoprotein cholesterol; HELNs, herb medicine-derived exosome-like nanovesicles; HFD, high-fat diet; HPCELNs, high PCELN-treated group; IL-1β, interleukin-1β; IL-6, interleukin-6; IL-18, interleukin-18; KEGG, Kyoto Encyclopedia of Genes and Genomes; LC3, microtubule-associated protein light chain-3; LDL-C, low-density lipoprotein cholesterol; LNPs, lipid nanoparticles; LPCELNs, low PCELN-treated group; MAFLD, metabolic dysfunction-associated fatty liver disease; MDA, malondialdehyde; MOD, model group; MPCELNs, middle PCELN-treated group; NC, normal control; NLRP3, nucleotide binding oligomerization domain-like receptor protein 3; NTA, nanoparticle tracking analyzer; Ox-mtDNA, oxidized mitochondrial DNA; Parkin, E3 ubiquitin ligase; PBS, phosphate-buffered saline; PC, *Poria cocos* (Schw.) Wolf; PCELNs, *Poria cocos*-derived exosome-like nanovesicles; PE, phosphatidyl ethanolamine; PINK1, PTEN-induced kinase 1; p62, sequestosome 1; ROS, reactive oxygen species; SOD, superoxide dismutase; TC, total cholesterol; TEM, transmission electron microscope; TG, triglyceride; TLC, thin-layer chromatography; TNF-α, tumor necrosis factor-α; UHPLC/MS, Ultra-high performance liquid chromatography–mass spectrometry; WATg, gonad white adipose tissue; WATi, inguinal white adipose tissue; 8-OHdG, 8-hydroxy-desoxyguanosine.
